# A Fat Fee

**Published:** 1889-07

**Authors:** 


A FAT FEE.—It is estimated that 50,000 medical practitioners would retire from the profession and rest from their labors if they could procure one such fee as that charged by Dr. Simmons, the physician of Samuel J. Tilden. The account was for 8 years practice, and amounts to $150,000.

